# Challenges and facilitators in treating unaccompanied young refugees with posttraumatic stress disorder in a dissemination trial: a qualitative study with psychotherapists

**DOI:** 10.1186/s13034-025-00873-w

**Published:** 2025-03-20

**Authors:** Flora Katrin Dietlinger, Barbara Kasparik, Johanna Unterhitzenberger, Laura Bebra Saupe, Rita Rosner

**Affiliations:** 1 Department of Psychology, Catholic University Eichstätt-Ingolstadt, Ostenstraße 25, 85072 Eichstätt, Germany; 2 Department of Social Sciences, Rosenheim Technical University of Applied Sciences, Am Industriepark 33, 84453 Mühldorf am Inn, Germany

**Keywords:** Unaccompanied young refugees, Psychotherapy, Challenges, Facilitators, Worries, Interpreters, Psychotherapists, Child welfare services, TF-CBT

## Abstract

**Background:**

Unaccompanied young refugees (UYRs) report high rates of post-traumatic stress, depression and anxiety, and low mental health service utilization. Studies have examined the experiences of psychotherapists and refugees in psychotherapy, focusing on barriers. Our stepped-care approach aims to reduce barriers through comprehensive support, such as training and case consultation for psychotherapists and interpreters, and treatment recommendations for UYRs.

**Methods:**

A qualitative design with semi-structured interviews was employed, with 20 psychotherapists, of whom 13 were females. All psychotherapists participated in the ‘BETTER CARE’ project, which included trauma-focused cognitive-behavioral therapy training and case consultations. We analyzed psychotherapists’ initial worries, challenges, and facilitators in treating UYRs with posttraumatic stress disorder, and compared the responses of completers’ and non-completers’ psychotherapists, following a mix of deductive and inductive coding.

**Results:**

Psychotherapists expressed worries similar to those documented in the literature on barriers (such as organizational challenges, emotional stress, and uncertainty about working with interpreters) prior to participating in the project. Major facilitators were the components offered by the project, such as online training, workshop and case consultations. In addition, support from the facility and caregivers and the provision of skilled interpreters who translated accurately and transparently, as well as patients’ treatment readiness and language proficiency, were seen as facilitators or, when lacking, as challenges. Completers’ psychotherapists were more likely to emphasize the positive aspects of the project, a positive therapeutic alliance and patients’ trusting relationship with the interpreters as facilitators. In contrast, non-completers’ psychotherapists were more likely to encounter structural difficulties, such as the lack of primary caregivers, greater distances, and grief symptoms among patients.

**Conclusions:**

Our findings indicate that enhancing the knowledge of psychotherapists, caregivers, and interpreters through specialized training is important for effective trauma treatment with UYRs. This training should result in increased patient readiness, caregiver support, and fostering a cooperative treatment environment, while also building a trusting relationship between patient, psychotherapist, and interpreter. As initial worries were largely unconfirmed, and completers’ psychotherapists benefited more from the projects’ offers, we recommend similar approaches.

*Trial registration*: German Clinical Trials Register DRKS00017453. Registered on 11 December 2019.

**Supplementary Information:**

The online version contains supplementary material available at 10.1186/s13034-025-00873-w.

## Background

Unaccompanied young refugees (UYRs) experience multiple stressors before, during, and after their flight [1, 2] and report high rates of post-traumatic stress disorder (PTSD; 4.6–43.0%), depression (2.9–61.6%), and anxiety (32.6–38.2%) [3]. Therefore, it is important that they receive appropriate interventions, as untreated posttraumatic stress symptoms (PTSS) can increase the risk of a wide range of physical and mental health conditions and chronic posttraumatic stress disorder [4, 5]. In light of the high needs, it is essential that evidence-based mental health care is available for this vulnerable group [6]. Trauma-focused cognitive-behavioral therapy (TF-CBT), developed by Cohen et al. [7], is recognized as an established treatment for PTSD in children and adolescents. The efficacy of TF-CBT has been demonstrated in meta-analyses and is recommended in international guidelines [8–10]. Studies have also demonstrated feasibility and efficacy in refugee and UYR populations [11, 12].

Despite the availability of treatment options, the use of mental health services among asylum seekers is low [3, 13–16]. Furthermore, studies on psychotherapy retention rates among UYRs are lacking, but a meta-analysis of adult refugees suggests a dropout rate of about 20%, which is comparable to non-refugee populations [17]. The difficulties of access and early dropout observed in this population can be attributed to several barriers. On the one hand, UYRs have complex needs, lack of trust in institutions and professionals, lack of awareness of mental health issues, lack of knowledge about the health care system, perceived discrimination, fear of stigma, and language barriers [16, 18–24]. On the other hand, they face additional structural barriers such as a lack of resources, legal restrictions/asylum status, and accessibility issues in terms of reaching psychotherapists and getting appointments [20, 22, 24, 25].

Additional barriers exist on the part of psychotherapists. Some psychotherapists are perceived as reluctant, lacking awareness, cultural competence and capacity, and have negative attitudes towards refugees, as reported by experts and refugees [16, 19, 22]. Previous research on psychotherapists’ experiences of working with refugees identified a mixed response to the use of interpreters [26]. The efficacy of interpreter assisted treatment has been demonstrated to yield outcomes that are comparable to those achieved in the absence of interpreters [26–28]. Psychotherapists themselves have reported several challenges during the psychotherapeutic process with refugees, including high bureaucratic and organizational effort, cultural challenges (different explanatory models of illness), different expectations of therapy, funding difficulties (intervention and interpreter), and communication and trust issues [29–32].

Given the existence of numerous qualitative studies examining psychotherapists' experiences in treating migrants or refugees (e.g. [29, 31, 32]) and the lack of studies on facilitators of psychotherapy with UYRs, this study is unique as it was part of a stepped care approach called ‘BETTER CARE’ [33]. This project is a multicenter dissemination and implementation trial that aims to improve the mental health of UYRs, to implement TF-CBT and to reduce barriers to psychotherapy utilization. Consequently, this study may provide new insights into additional relevant factors. Therefore, we sought to assess whether the concerns reported by psychotherapists prior to their participation in the ‘BETTER CARE’ project were consistent with barriers documented in the literature. We also examined reported facilitators and challenges after the primary barriers were removed by the project design, and whether these differed between psychotherapists whose patients completed the TF-CBT and those who terminated prematurely.

## Methods

### Study context

The data were collected as part of the multicenter, cluster-randomized controlled trial ‘BETTER CARE’ [33]. The trial and all subprojects were approved by the ethic committees of Ulm University (No. 243/19) and Catholic University of Eichstätt-Ingolstadt (No. 004-19). The main aim of the trial is to compare a stepped-care model, including the preventive group intervention “My Way” [34] and TF-CBT [7], with treatment as usual. Participating UYRs were recruited through Child and Youth Welfare Services (CYWS) facilities via welfare lists. A mental health screening was conducted on each adolescent to assess the presence of PTSS, depression, and anxiety symptoms. If cut-off scores were exceeded, treatment recommendations were made for either My Way (PTSS subclinical) or TF-CBT (PTSS clinically significant). The CYWS facilities were provided with lists of nearby TF-CBT-trained psychotherapists and interpreters who were themselves participating in the BETTER CARE project. Although interpreters were trained as part of the project, none of them accompanied treatments in this subsample due to long distances or other reasons of unavailability.

Inclusion criteria for psychotherapists in the project were: licensed child and adolescent psychotherapist or psychological psychotherapist; willingness and written informed consent to participate and willingness to treat up to three UYRs with PTSS. As a first step, the psychotherapists did the German TF-CBT Web, a web-based training programme (http://tfkvt.ku.de) which is enhanced with culturally tailored materials. It has previously shown effectiveness and acceptance among psychotherapists [35]. Afterwards, they received a two-day online live workshop, led by a TF-CBT trainer, focusing on treatment methods and special considerations for working with UYRs. This included the importance of cultural aspects in TF-CBT, such as maintaining an open attitude of not knowing and navigating between cultures, as well as psychoeducation on confidentiality and psychotherapy in general, and the involvement of interpreters and professional caregivers as a bridge to the facility. Psychotherapists treating project participants were required to participate in bi-weekly live online case consultations led by TF-CBT trainers. Case consultations were conducted in same groups of two to six psychotherapists. Once the psychotherapists had completed their first case, they were eligible to participate in the follow-up interview, the period between the workshop and the interview were *M* = 333.94 days (*SD* = 170.74, range: 136–650 days, 2 missings). To participate in the interview, they had to sign an additional consent form.

### Procedure

The interviews were conducted between September 2021 and February 2024. Participants did not receive any form of compensation for the interview. The duration of the interviews was between 22 and 54 min. All interviews were documented through audio recordings and subsequently transcribed word-by-word. Any information in the interview transcripts that could potentially identify the participants or UYRs was removed to ensure anonymity. The individual online interviews were conducted by BK and FD, who both held master’s degrees in clinical psychology, using the online video platform ‘ZOOM’ with end-to-end encryption. Both interviewers were research assistants in the ‘BETTER CARE’ project and were responsible for recruiting psychotherapists and organizing the training and case consultations, but they were not trainers/supervisors.

### Participants

The sample consisted of 20 psychotherapists, of whom 13 were female and 13 had previous experience in treating UYRs (Table 1). All psychotherapists had been licensed psychotherapists for at least one year (*M* = 8.70; *SD* = 5.96, range: 1–23 years). 18 participants were child and adolescent psychotherapists. Only two were psychological psychotherapists, one of whom also held an additional license as a child and adolescent psychotherapist. In Germany, psychological psychotherapists are professionals who, after obtaining a degree in psychology, have completed an extensive postgraduate training program in psychotherapy, specializing in the treatment of either adults, youth, or both. A total of 17 psychotherapists specialized in cognitive-behavioral therapy and three specialized in psychodynamic psychotherapy. Seven psychotherapists had only one or more non-completers and no ongoing or successfully completed TF-CBT, while six psychotherapists had only one or more psychotherapy completers and no ongoing or non-completed TF-CBT. The psychotherapists treated in total 33 study patients. Consisting of five females and 28 males, with ages ranging from 13 to 19 years (*M* = 16.18, *SD* = 1.17). Among them, 32 patients were residing in CYWS facilities, while one patient stayed in a so-called supervised independent living, where youth receive less intensive care but remain connected to the CYWS facility. All patients underwent a two-step diagnostic procedure. Following referral due to elevated screening scores, each patient received a clinical diagnosis by the psychotherapist. In total, 22 patients were diagnosed with PTSD alone; six with PTSD and a moderate depressive episode; two with PTSD and a mild depressive episode; and one with PTSD and a chronic pain disorder. The diagnostic information for two patients is missing. The data do not show a clear distribution that would indicate that either psychotherapists or patients are predominantly located in urban or rural areas. Since none of the accompanying interpreters participated in the BETTER CARE training, we do not have socio-demographic data on the interpreters.

**Table 1:** Participants’ Sociodemographic characteristics

ID	Gender	Educational background	Theoretical background	Experiences since licensure (years)	Previous experience in treating UYRs	No of treated UYRs in the project		
						Dropout	Completed	Ongoing
T1	M	Psychology, Social Pedagogy	CBT	8	Yes	2	0	0
T2	F	Social Pedagogy	PP	23	No	1	1	0
T3	F	Pedagogy	CBT	5	Yes	1	1	0
T4	M	Social Work, Social Pedagogy	PP	8	No	0	3	0
T5	F	Psychology	CBT	20	Yes	1	0	0
T6	M	Social Pedagogy	CBT	8	Yes	2	1	0
T7	M	Education	CBT	6	Yes	0	2	0
T8	F	Social Work	CBT	6	No	0	1	1
T9	F	Social Work	CBT	6	No	0	1	0
T10	F	Social Pedagogy, Social Work	PP	18	Yes	0	1	1
T11	M	Pedagogy	CBT	6	Yes	1	0	0
T12	F	Social Pedagogy	CBT	7	Yes	1	0	1
T13	F	Social Work	CBT	4	No	1	0	0
T14	F	Pedagogy	CBT	5	Yes	0	1	0
T15	F	Social Pedagogy	CBT	1	Yes	0	1	0
T16	F	Social Work	CBT	9	Yes	0	2	0
T17	M	Psychology	CBT	4	No	1	1	0
T18	F	Psychology	CBT	11	No	1	0	0
T19	F	Psychology	CBT	16	Yes	1	0	0
T20	M	Pedagogy	CBT	3	Yes	1	0	0

M: Masculine; F: Feminine; CBT: Cognitive Behavior Therapy; PP: Psychodynamic Psychotherapy

### Interview

The interviews followed a semi-structured format, following an interview guide developed specifically for this study (see Additional file 1). The interview questions were designed based on the knowledge of treatment challenges as found in the literature (e.g. [36]). The preliminary interview guide was subjected to a review process involving team members, all of whom were licensed psychotherapists with expertise in treating UYRs, and qualitative research. This resulted in an adjusted interview guide, based on the feedback received. The final version was piloted in a test interview with a fellow psychotherapist and included questions about previous experience in treating traumatized children and youth, and traumatized UYRs. It also inquired about experiences with the ‘BETTER CARE’ stepped-care approach, specifically the facilitators and challenges relevant to the implementation of TF-CBT with UYRs, treatment fidelity, and sustainability of the training and study. All interviews were conducted in German.

### Data analysis

The data were transcribed using the software “amberscript”, the transcripts were then proofread and corrected by the team members and analyzed according to the focused interview analysis by Kuckartz and Rädiker [37]. After an initial familiarization with the content of the transcripts, the coding process combined inductive and deductive approaches, supplemented by analytical memos to discuss unclear expressions. Two authors developed a preliminary codebook from four interviews, which was refined through ongoing analysis with input from a third author, reaching consensus on codes after review and a calculated inter-rater reliability of *k* = 0.78. Discrepancies in coding were discussed and resolved, resulting in a defined code list that was analyzed using MAXQDA24. Codes that were mentioned only once were grouped under the code “other” and reported with two examples each. The frequencies were reported according to the function “Code Frequencies—Valid Percent” in MAXQDA 24. The frequencies of specific codes within the dataset were then examined in relation to the total number of valid codes, excluding interviews that did not address this topic. A comparison was made between the facilitators and challenges mentioned by psychotherapists with patients who completed TF-CBT (completers' psychotherapists) and those with patients who prematurely terminated TF-CBT (non-completers' psychotherapists). This was based on the rule of “counting each hit in a document only once” and a minimum difference of two mentions between the groups in the number of codes.

## Results

Psychotherapists who had experience in working with UYRs mentioned the following experiences: referral through the CYWS institution/school context, challenging life circumstances (such as traumatic experiences in the host country that prompted them to seek treatment), and uncertainty in treatment and dealing with cultural differences. The worries psychotherapists expressed prior to their involvement in the project were mainly related to structural barriers, such as organizational difficulties and uncertain residency status; personal concerns such as high emotional strain and cultural competence; and patient-related issues, such as poor language skills, unreliable attendance, and severity of psychopathology, as seen in Additional file 2.

The study revealed that participants identified various factors, including project-related, structural, personal, patient-, and interpreter-related aspects, as either facilitating or challenging the implementation of psychotherapy with UYRs. Table 2 provides an overview of the categories and subcategories identified in the interviews regarding the facilitators and challenges that emerged. In the following text, all categories that were identified during the analysis are presented in italic font. In Additional file 3, Add3-Table 1–Table 5 present all results with codes, frequencies, and examples.

**Table 2:** Facilitators and challenges in working with UYRs

Facilitators	Frequency	Challenges	Frequency
**Project-related**		**Project-related**	
		No challenges^b^	8 (44.4%)
Case consultations^a^			
General effectiveness	12 (60.0%)		
Group-based learning	8 (40.0%)		
Increases adherence	7 (35.0%)		
Enables flexibility within fidelity	5 (25.0%)		
Reflecting about patient	5 (25.0%)		
Supervising person as competent contact person	4 (20.0%)		
TF-CBT Workshop^a^			
General effectiveness	4 (20.0%)		
Conveys important knowledge	4 (20.0%)		
Structure of the workshop	2 (10.0%)		
Both case consultations and workshop^a^	5 (25.0%)		
Material^a^		Material^b^	
General availability of material	12 (60.0%)	Material not adequate	3 (15.0%)
Availability of worksheets	5 (25.0%)		
Availability of translated materials	5 (25.0%)		
German TF-CBT Web^a^		German TF-CBT Web^b^	
General effectiveness	4 (20.0%)	Too extensive	2 (10.0%)
Video examples	3 (15.0%)		
Repeated use possible	2 (10.0%)		
Flexibility	2 (10.0%)		
Manualized and evidence-based treatment^a^	9 (45.0%)	Documentation^b^	7 (35.0%)
Provision/Funding of interpreters^a^	8 (40.0%)	Lacking information on organizational aspects^b^	4 (20.0%)
Financial compensation for psychotherapists^a^	8 (40.0%)	Knowledge decay^b^	2 (10.0%)
Preparation and Initiation of treatment^a^	7 (35.0%)		
Availability of contact persons^a^	5 (25.0%)		
Digital implementation^a^	3 (15.0%)		
**Structural**		**Structural**	
CYWS facility aspects^c^		CYWS facility aspects^a^	
Supporting/accompanying the treatment sessions	7 (38.9%)	Lacking clear primary clearly responsible caregiver	7 (35.0%)
High treatment compliance from caregivers/facility	8 (44.4%)	Lacking therapy compliance from caregivers/facility	3 (15.0%)
Supportive caregivers in everyday life	5 (27.8%)	Caregiver-imposed outcome pressure	2 (10.0%)
Collaboration and exchange between caregivers and psychotherapist	3 (16.7%)	Lacking caregiver/conjoint sessions	4 (20.0%)
Knowledge about PTSD/psychotherapy	2 (11.1%)	Lacking knowledge about PTSD/psychotherapy	3 (15.0%)
Facilitating transportation^c^	2 (11.1%)	Logistical access issues	4 (20.0%)
Availability of interpreters ^c^	9 (50%)	Long distance between facility & psychotherapy ^a^	7 (35.0%)
Use of supplemental materials^c^	4 (22.2%)	Difficult time coordination^a^	8 (40.0%)
Location of the practice^c^	2 (11.1%)	Increased effort^a^	6 (30.0%)
		Technical issues^a^	4 (20.0%)
		Lacking clear responsibility from Youth Welfare Office^a^	4 (20.0%)
		Unreliability of public transportation^a^	2 (10.0%)
**Personal**		**Personal**	
Good therapeutic alliance^d^	6 (46.2%)		
Openness to treat UYRs^d^	3 (23.1%)		
**Patient-related**		**Patient-related**	
Treatment readiness^e^	6 (54.5%)	Lacking treatment readiness^f^	5 (33.3%)
Language proficiency^e^	2 (18.2%)	Lacking language proficiency^f^	9 (60.0%)
		Concerns regarding family^f^	6 (40.0%)
		Complex daily challenges^f^	4 (26.7%)
		Grief^f^	2 (13.3%)
		Lacking educational background^f^	2 (13.3%)
**Interpreter-related**		**Interpreter-related**	
Precise word-for-word translation ^f^	6 (40.0%)	Lacking word-for-word translation ^g^	6 (60.0%)
Transparency^f^	6 (40.0%)	Own therapeutic needs^g^	4 (40.0%)
Trusting bond with patient^f^	6 (40.0%)	Relationship with patient too close^g^	3 (15.0%)
Caring/likeable interpreter^f^	6 (40.0%)	Remote interpreters ^g^	3 (15.0%)
Experienced/Trained interpreter^f^	5 (33.3%)	Interpreters wish to act as co-therapist^g^	2 (10.0%)
Cultural mediator^f^	4 (26.7%)		
Language mediator^f^	3 (20.0%)		
Remote interpreter^f^	3 (20.0%)		
Long-term continuity^f^	2 (13.3%)		

^a^Interviews with code *n* = 20

^b^Interviews with code *n* = 18

^c^Interviews with code *n* = 19

^d^Interviews with code *n* = 13

^e^Interviews with code *n* = 11

^f^Interviews with code *n* = 15

^g^Interviews with code *n* = 10

### Project-related facilitators and challenges

Project-related aspects were most frequently reported as facilitators, as shown in Add3-Table 1 (see Additional file 3). First, the biweekly live online *case consultations*, which were funded by the project, were identified as the most important facilitator due to their dual efficacy in providing assistance and fostering provided group-based learning. In addition, the case consultations were perceived as enhancing and facilitating adherence because “[…] she [the supervisor] was always able to give good tips on what one can do to perhaps find one’s way back a little bit.” (T4). It also allowed for flexibility within fidelity and was a space “[…] where you can reflect on patients again […]” (T18). Participants also expressed appreciation for the supervisors' expertise and ability to help when needed. Other facilitators of the case consultations were, for example, collegial interaction and appreciation by the supervisors. The only negative aspect of case consultations was the lack of opportunities for peer interaction. “That is, not only the individual communicates with the [supervisor], but that the others can also participate a bit… if they have questions or if they have suggestions or a feeling about it. That it's more of a group setting, instead of just being spectators while two talk to each other. I would wish for that a bit more, or I could express it as a point of criticism.” (T4).

Second, another important component was the *TF-CBT workshops*, which were not only mentioned as being generally effective but, according to the psychotherapists, also provided important knowledge about the manual, as one has “[…] now experienced in the workshop what it can look like.” (T4). They also emphasized the good structure of the workshop. Other facilitators were, for example, the opportunity to ask questions and the online implementation. “And then the workshop. It was fantastic. First of all, online, and then also just the two of us. I mean, it doesn't get much better than that.” (T9). Participants did not mention any challenges related to this. For five people, *both the case consultation combined with the workshop* was the most helpful aspect of the study.

Third, the *provided materials* were facilitating. The general provision, and especially the availability of worksheets and translated materials, were perceived as beneficial for facing challenging circumstances: “[…] whenever I thought I was floundering, I thought, okay, then I'll cling to what the worksheets provide.” (T16). Some psychotherapists stated that the materials were not effective, for example, in a situation where the patient “[…] was already 20 years old and knew a lot, I rather worked with materials from the adult sector or with materials I produced myself.” (T5).

Fourth, the *German TF-CBT Web*, which the participants were required to complete as a preliminary step, served as a facilitator in the training process, providing not only knowledge but also video examples and an initial overview that could be revisited as often as necessary. “So, for almost every session, before every module, I reviewed and read through the module again. Also, I watched one or the other video again.” (T4). The accessibility and flexibility of the website, coupled with its comprehensive content, made it an ideal learning resource. The other facilitator mentioned was that it gave a first insight into conducting TF-CBT. The challenge mentioned was that the program was too extensive, and one psychotherapist further stated, “I'm not much of an online learner. It annoys me.” (T18).

In addition to the components provided by the study, participants also recognized the benefit of using a *manualized and evidence-based treatment*. “Okay, so this is, of course, a manual, so to speak. There are aspects, so to speak. It's about doing it just like that. And that's exactly it, no more and no less. So that is, that is, I find it quite okay again, because then, both I and the therapist, uh the patient knows what he is getting, so to say.” (T1). The *provision and funding of interpreters* by the project, as well as the *financial compensation for the psychotherapists*, were seen as beneficial. In addition, the *preparation and initiation of the treatment* “[…] through the making of contact and assignment of patients, this greatly eases the situation.” (T5) were mentioned. The psychotherapists also appreciated the *availability of contact persons* that “[…] you could always ask [via email] if anything was missing and certainly the contact with the study center as well.” (T19). The *digital implementation* of all aspects of the study was an additional factor that facilitated the implementation. Other facilitators mentioned were for example, the spontaneous and uncomplicated study participation and “[o]f course, without ‘BETTER CARE’ it wouldn't have happened, and it's somehow, I would say nice, to be part of such a network. So, I benefit from it too and everything that is done about it.” (T15).

Eight psychotherapists reported no challenges related to the study, and two psychotherapists did not respond. In addition to the *documentation effort*, another challenge was the *lack of information on organizational aspects* and *knowledge decay* between the training and seeing of the first patient. “I just found it a bit difficult until the first patients arrived. Naturally, you then slip back into your daily routine, because, yes, I also treated many patients and then that part slips away again. I found that a shame, because it then required more effort from me, so to say.” (T2). Other challenges mentioned included a lack of support from study staff members for structural problems and inappropriate diagnostic tools.

In conclusion, the main project-related facilitators were case consultations and the availability of materials that was each mentioned by 60% of the participants, followed by manualized and evidence-based treatment (45%). The main obstacle was the documentation (38.9%).

### Structural facilitators and challenges

Structural aspects were reported as both facilitators and challenges, with some codes overlapping as can be seen in Add3-Table 2 (see Additional file 3). Most of the structural aspects mentioned were related to the *CYWS facilities*. As facilitators, the psychotherapist mentioned when “[…] always the same primary caregiver [was] involved […]” (T11), as well as high treatment compliance by the caregivers and/or the facility. When this was not the case, it was also mentioned as a challenge. Furthermore, the provision of caregiver assistance with activities of daily living was identified as a crucial element, “[…] because they had a good caregiver network, I didn't have to worry about things like school, residence permits, or anything else, but I could focus on the therapy.” (T16). Collaboration, caregiver-therapist sharing, and knowledge about PTSS and psychotherapy were helpful; conversely, absent caregivers or shared sessions and knowledge gaps were challenging. On the one hand, facilitating transportation was helpful, “[although] it often didn't work out well for them to participate in person, but they really made an effort to ensure that he… that he was taken to his appointments, so that he was picked up. They always registered in advance. So, the facility was very committed, and I believe it also made things easier.” (T3). On the other hand, logistical access issues when caregivers were unable to provide transportation, were relevant challenges. Other facilitators related to the CYWS facility were e.g. when “[they] […] had a very clear information system in the house, that they did it like hospitals and kept the files online, and no matter who you talked to, they knew exactly what had been discussed with the previous person. Which is not a matter of course with the facilities.” (T7) and less personal fluctuation. In addition to the aforementioned challenges, psychotherapists identified pressure from caregivers as a significant difficulty, as “[…] [they] hope for quick help from the therapist, kind of like this'fix it quickly so things run smoothly'.” (T18). Other facility-related challenges included the need to work in shifts, absenteeism, and the difficulty of reaching caregivers and patients by phone.

In addition to the CYWS–related aspects, the *availability of interpreters* emerged as an important facilitator, as was the *use of supplemental materials* and the *location of the practice* when “[…] the practice is perhaps relatively central. The thing with the train station, I believe, is also quite good […]” (T1). Other structural facilitators were, for example, the uncomplicated approval of the treatment by the health insurance: “[t]he approval was really quick and completely hassle-free, and for the first one, he then started training, and so the youth welfare office practically handed it over to the health insurance. And then I just got the remaining hours approved by the health insurance. That was totally easy, totally relaxed. It went really well.” (T3) and offering double lessons at the end of the day.

Other challenges besides the CYWS facility were the *distance between the facility and the psychotherapeutic practice*. In addition, psychotherapists’ *increased effort* and a difficult *time coordination* were identified as challenging, for example, “[…] to synchronize the interpreter's schedule and mine, and if it didn't work out on a certain day, we usually didn't find any alternative dates. Because we were both fully booked, and it just didn't work out.” (T7). Those who provided digital treatments during the Covid-19 pandemic also encountered *technical problems*. For a significant number of patients, the cost of psychotherapy was covered by the Youth Welfare Office, and in this area the psychotherapists mentioned a *lack of responsibility*. This was explained as follows: “I have to clarify it in advance. But nothing comes back from there. Like, how often I have called and sent emails. In every phone call, I'm put off, referred to someone else. It's really, really difficult.” (T4). Finally, the *unreliability of the public transportation* was also a challenge. Other challenges mentioned were, for example, the lack of clear responsibility of the legal guardian and uncertainty about the residence status.

One psychotherapist did not mention any structural facilitators. The most important facilitators were availability of interpreters (50%), high compliance of caregivers/facilities (44%), and when caregivers supported/accompanied treatment sessions (39%). The main challenges were time coordination (40%), a lack of clear responsible caregivers (35%), and long distance between facility and psychotherapy (35%). The most important facilitators and challenges were focused on the CYWS facility and its caregivers and their commitment to psychotherapy.

### Personal facilitators and challenges

Add3-Table 3 (see Additional file 3) shows that personal facilitators or challenges were rarely mentioned. Only 13 psychotherapists named personal facilitators, most commonly the ability to form a *good therapeutic working alliance*. Furthermore, participants indicated that it was beneficial to be *open to treating UYRs*, as “I also like treating refugees! But it might make things a bit easier, indeed.” (T1). Other facilitators mentioned were, for example, when “[someone] already knew the caregiver before ‘BETTER CARE’ and the interpreter as well, and yes, it was just a good foundation that [they] had already established beforehand.” (T12).

Personal factors that posed a challenge to the treatment of UYRs were rarely mentioned. Only five psychotherapists reported such occurrences, and no recurring themes emerged. Therefore, we provide selected examples, including a wrong impression of the need for a interpreter and impatience. One psychotherapist articulated her experience as follows: “So, I can be a bit impatient sometimes, I then have to hold myself back and say, maybe this isn't the topic for today after all. We need to take a step back again.” (T15).

### Patient-related facilitators and challenges

The psychotherapists mentioned more patient-related challenges than facilitators, as can be seen in Add3-Table 5 (Additional file 3). The main themes were *therapy readiness* and *language proficiency*. These were identified as both facilitators and challenges, depending on their presence or absence. One psychotherapist explained the lack of treatment readiness as follows: “We then revisited the symptoms, yes, I believe, this concept of allowing oneself to be helped was not yet acceptable to him. He was not at that point yet.” (T18). Another psychotherapist described the lack of language skills in this way: “I actually found the language barrier difficult, even though, of course, a interpreter could have been used, but there was no acceptance on the part of the patient, at least at that time.” (T13). Other patient-related facilitators included, for example, being likeable and cognitively talented.

Additional challenges were *concerns about the family*, as “[…] he somehow needs certainty over this'my family is coming or not coming' in order to either deal with the fact that they are not coming, or if they do come, then the traumatic issues will resurface for him. At the moment, all of this doesn't come up at all, it has no meaning for him.” (T3). Furthermore, *complex daily challenges* such as “[…] the turbulence of their everyday lives. Yes. Youth welfare and trauma have the potential to make everyday life unstable.” (T20) hindered a stable therapeutic process. Psychotherapists mentioned that for some patients, *grief* was a more prominent factor, impeding their ability to process the trauma. Finally, a *lack of educational background* made it difficult for the patients to complete the worksheets. Other mentioned patient-related challenges were, for example, the presence of a rigid mindset and stuttering.

Nine psychotherapists did not mention any facilitators and five did not mention any challenges. Overall, therapy readiness (facilitator: 54.5%, challenge: 33.3%) and language proficiency (facilitator: 18.2%, challenge: 60.0%) were the most frequently mentioned factors that can facilitate or hinder psychotherapy with UYRs.

### Interpreter-related facilitators and challenges

As can be seen in Add3-Table 5 (see Additional file 3), the concept of *precise, word-for-word translation* was perceived as a facilitator, while its absence was perceived as a challenge. A *transparent* communication with psychotherapists about the interpreters’ inability to provide word-for-word translation was seen as beneficial, for example when “[…] he also always asked again and said, when he mentioned: well, you know, this and that in the language is difficult to explain. May I, so, he also asked, even if it went beyond the translation […]” (T18). Similarly, *experienced and trained interpreters* were seen as advantageous. A *trusting bond* including “[…] the human aspect, meaning that one could tell that the young people also liked talking with this interpreter. That was also very important. And they actually treated him like an uncle.” (T7). Nevertheless, some psychotherapists noted that a *too close relationship* was difficult for the treatment, as one psychotherapist had “[…] the impression that some young people tend to bond more with the interpreter than with me.” (T16). The *likeability and care* exhibited by interpreters facilitated the treatment for example when “[…] she participated, truly participated with her heart […]” (T4). In addition to serving as language mediators, the interpreters also served as *cultural mediators*, which “[…] was positive, clearly, that was also a form of cultural mediation alongside the linguistic, I would say.” (T17). The use of *remote interpreters* was perceived as both a facilitator and challenge. One psychotherapist described “[…] the switch to the online interpreter was better because, due to, because the computer was there, it was a bit more in the background.” (T11) while another one saw that “[t]he difficult part was that he was only available by phone, so to speak. I didn't find that very helpful. I do think it's good to be able to see the person. So, it wasn't good that he didn't manage to do it with a phone [means: video].” (T1). In addition, the *interpreter's long-term continuity* in the process, coupled with his involvement in other contexts, proved to be advantageous in terms of acquiring more information about the patient. Other interpreter-related facilitators mentioned were, for example, flexibility and reliability.

Besides the challenges described above, when interpreters had their *own therapeutic needs* and when they wanted to *act as a co-therapist* for example “[…] when they did not just perform their task but tried to act therapeutically themselves or became too emotional […]” (T6) was seen as a challenge to the treatment. Other challenges mentioned were, for example, short notice cancellations and multiple interpreters for one patient.

With regards to the role of facilitators in working with interpreters, five participants did not respond, while only ten psychotherapists responded to the question of challenges. In summary, it is evident that the priority for psychotherapists is the accuracy (facilitator: 40.0%, challenge: 60.0%) and transparency (facilitator: 40%) of the translation. Although establishing a trusting bond with patients was seen as beneficial (40.0% each), maintaining a balance is crucial, as a bond that is too close was seen as a challenge (15.0%).

#### Comparison of completers’ psychotherapists vs. non-completers’ psychotherapists

A comparison of the facilitators and challenges mentioned by six completers’ psychotherapists versus seven non-completers’ psychotherapists indicated the following.

##### Project-related aspects

Completers’ psychotherapists were more likely to emphasize the effectiveness of case consultations (83.3% vs. 26.8%) and the provision of interpreters (66.7% vs. 14.3%). Only completers’ psychotherapists mentioned the usefulness of worksheets (33.3%) and online learning videos (33.3%). Documentation challenges were more commonly reported by completers' psychotherapists (66.7% vs. 14.3%). Non-completers' psychotherapists were more likely to emphasize the benefits of manual and evidence-based methods (57.1% vs. 33.3%).

##### Structural aspects

Completers’ psychotherapists perceived supplemental materials as facilitating (50%) and lacking clear responsibilities from Youth Welfare Offices (33.3%) as challenging. These issues were not addressed by non-completers’ psychotherapists. Non-completers’ psychotherapists were more likely to report unclear responsibility of primary caregivers (42.9% vs. 16.7%), distance issues (57.1% vs. 16.7%), and additional effort reported (42.9% vs. 16.7%).

##### Personal aspects

Completers’ psychotherapists reported the beneficial impact of a positive therapeutic alliance (50.0% vs. 14.3%) more often, while only non-completers’ psychotherapists indicated that willingness to treat UYRs was a significant factor (28.6%). There were no notable differences between challenges.

##### Patient-related aspects

There was no notable difference between the facilitators. Only completers’ psychotherapists reported a lack of educational background as a challenge (33.3%), while only non-completers' psychotherapists reported grief as a significant challenge in implementing TF-CBT (28.6%).

##### Interpreter-related aspects

Only for completers' psychotherapists, a trusting relationship between the interpreter and the patient (50%) and long-term continuity (33.3%) were important factors. No remarkable differences in challenges were noted.

## Discussion

This study contributes to the current literature by exploring the experiences of psychotherapists working with UYRs in a dissemination and implementation trial. It further provides new insights into the facilitators of trauma treatment in a refugee population, after efforts have been made to address several care-related barriers as part of the ‘BETTER CARE’ project.

We aimed to examine whether psychotherapists reported the *same worries before participating* in the project as reported in the literature. We found partial evidence, especially for structural and psychotherapist-related worries, such as organizational and residency issues [22], the involvement of interpreters [24], cultural competence [19, 25], and emotional burden for the psychotherapist [38]. Patient-related worries were also reported, such as language barriers [16, 22, 30] and unreliable attendance, which may be due to different cultural concepts of time [24]. However, a quarter reported no worries, indicating openness and low prejudice towards psychotherapy with UYRs in our sample.

Because the *project* attempted to address primary structural barriers, the study found that initial worries largely did not re-emerge as challenges, with psychotherapists facing mainly coordination issues, but not the anticipated emotional distress or problems related to interpreters and cultural competences. Patient-related worries, particularly language skills, persisted, while issues such as attendance and severe psychopathology were less prominent. Thus, it appears that the inclusion of resources such as the German TF-CBT Web, workshops, and case consultations significantly facilitated the delivery of psychotherapy to UYRs. Therefore, we can conclude that psychotherapists who were interested in learning evidence-based treatment methods and treating UYRs responded positively to the combination of an online learning platform and a workshop, although a potential bias in the interpretation of the results should be considered, as those who were not interested in online training and (UYR) trauma treatment did not participate in the project. The results align with research highlighting the value of formal training for positive treatment outcomes [29, 39] and the combination of a variety of strategies [40]. In addition, psychotherapists highlighted the benefits of preparing and organizing psychotherapy through the project, in addition to support by the project staff. This included initiatives such as: a brief training for a mental health coordinator to act as a contact person within the CYWS facility, making phone calls to determine whether UYRs recommended for treatment were interested in psychotherapy, and if so, making further phone calls to verify if these adolescents received a psychotherapy slot. The project is considered a successful approach, as evidenced by the fact that eight out of 18 participants reported no challenges associated with the project. The primary challenge identified, documentation, is likely due to the extensive project-specific paperwork required for a detailed evaluation and thorough understanding of the treatment process. Challenges related to the TF-CBT treatment approach could be addressed by providing organizational information about common issues related to psychotherapy funding and interpreters within the learning platform, and by providing separate materials for younger children and adolescents.

From a *structural perspective*, psychotherapists emphasized the importance of caregivers supporting and/or accompanying psychotherapy, which is also an integral part of the TF-CBT manual [41]. This is particularly important because, in addition to providing emotional and practical support, they are relevant as models of resilient coping mechanisms and protection against future harm [41]. The psychotherapists in our study agreed on this part with the manual that points out the immense impact of caregiver inclusion during the psychotherapeutic process. In addition, a supportive infrastructure within the facility, which includes not only facilitating transportation to psychotherapists, but also caregiver compliance and knowledge about PTSS and psychotherapy, was seen as critical to UYRs' treatment attendance and compliance. McGuire et al. [42] identified several potential reasons for the lack of caregiver support and involvement, including limited staff availability for one-on-one support, placement nature that occasionally prevents caregiver presence, instability within the support network, and scenarios where the youth preferred to exclude their caregivers. Compounding these challenges, the location of the psychotherapeutic practice, and the distance between it and the facility, along with unreliable public transportation, were also highlighted as significant challenges in our study, suggesting remote or outreach psychotherapy as an alternative. Remote psychotherapy received mixed reviews in our study; some psychotherapists highlighted problems stemming from the lack of personal contact and a secure space, which are vital for effective treatment. This issue was particularly pronounced when psychotherapy was conducted solely via online platforms during the Covid-19 pandemic, while the patients were often located in shared rooms. This created situations in which they might avoid sharing traumatic events, especially since many of these experiences are associated with feelings of guilt and shame. Therefore, an outreach approach, in which psychotherapists meet patients at home or at a location of the patient's choice, may reduce barriers and facilitate caregiver involvement [43]. Additionally, under such circumstances, psychotherapists could ensure that psychotherapy takes place in a safe space for the patient. If this is done by psychotherapists who are interested and trained in working with UYRs, it could also help facilitating the process of bringing them into psychotherapy, especially as the willingness of outpatient psychotherapists in Germany to treat refugees is lower than that of non-refugee patients [44].

The psychotherapists interviewed were less likely to mention *personal factors*. The most important factor was the ability to build a strong relationship with patients. This finding is consistent with studies indicating that a strong therapeutic relationship is an important component of TF-CBT, leading to reduced PTSS [45], with the alliance being particularly important at mid-treatment. Ormhaug et al. [45] also found that a positive alliance also led to more efficient treatment, with fewer sessions. In addition to facilitating psychotherapy, a positive alliance may also reduce dropout rates among refugees, as shown in a recent review with refugees [17]. To strengthen the alliance, studies by Colucci et al. [24] and Mirdal et al. [36] further emphasize the need for mental health professionals to use presence-focused, adolescent-friendly approaches and to provide psychoeducation. Psychoeducation plays an important role in the process of establishing a strong alliance with refugee patients, as they often come from a different cultural background, have a different explanatory model and different expectations of psychotherapy [29]. Promoting psychoeducation can help to build trust and safety by providing clear information.

The most important *patient-related factors* influencing the psychotherapeutic process were readiness for psychotherapy and language skills. The significance of these factors has also been reported by previous research [16, 22, 30] and reflects prior worries articulated by psychotherapists in the current study. The findings indicate that the project team's involvement in the preparation and initiation of psychotherapy was perceived positively by psychotherapists, leading to patients who were better informed and had undergone a pre-selection process. In fact, each participant received a letter post-screening that included treatment recommendations based on their sum scores. Based on this, utilizing digital screening tools that offer automatic treatment recommendations and ideally, the contact details of trained psychotherapists nearby, is advisable. In addition, knowledge of psychotherapy and PTSS among CYWS staff was seen as beneficial. Therefore, it appears that staff training is important to enable them to provide psychoeducation to UYRs and to increase readiness for psychotherapy. Summarizing the results, the information and screening approach implemented in the ‘BETTER CARE’ project can be considered successful, especially as the commonly discussed mental health illiteracy among refugees [29] was not reported as a challenge in this study. Patient-related challenges included family concerns or daily challenges. These are known to be important factors that also influence well-being [46], underlining the need to address them and achieve early improvements in psychotherapy, as noted by Mirdal et al. [36] and Colucci et al. [24]. Although language proficiency is not mandatory, it greatly facilitates effective psychotherapy. However the lack of b language skills were experienced as challenging by psychotherapists in our study.

Given the importance of language proficiency, it’s not surprising that the provision of trained *interpreters* was mentioned several times as being helpful. A trusting, collaborative relationship between interpreter, patient and psychotherapist was reported not only by psychotherapists, but also in a study that included the views of all three parties [36]. For psychotherapists, accurate translation skills and transparency are important, but so are the interpreters’ soft skills such as building trust with the patient and showing empathy. It is noteworthy that psychotherapists in our study also indicated that an overly close bond could be problematic. This finding also aligns with Mirdal et al. and Vivino et al. [36, 47], both of whom reported interpreter involvements that exceeded western expectations regarding the professional distance. This highlights possible cultural differences and conflicting expectations regarding the role of the interpreter. As the majority of patients and their interpreters originate from collectivist societies where community involvement is more important, group identity may be incompatible with the “neutrality” required by individualistic psychotherapists [24, 48]. To reduce potential problems, it is suggested that a training program for interpreters be implemented, that focuses on translation in trauma-focused psychotherapy, particularly with regard to the rationale behind the trauma narrative. This knowledge could lead to more accurate translations and improve the quality of collaboration within the therapeutic triad [49]. Finally, the results of the interviews show that the inclusion of interpreters is advantageous because interpreters can foster a trusting relationship and are able to show empathy and provide a comfortable and safe environment for the patient, which is essential in the therapeutic context. In addition, they serve not only as language mediators, but also as cultural mediators, enhancing communication in ways that artificial intelligence translation applications cannot replicate. Moreover, the use of most AI translation tools is not advisable as they often process data externally and risk data breaches. Comparing answers from *completers’ psychotherapists and non-completers’ psychotherapists* regarding* facilitators*, revealed that completers’ psychotherapists benefited from resources such as case consultations, worksheets, videos, and interpreters funded and provided by the project. These psychotherapists also formed stronger alliances with patients and trusted long-term relationships with interpreters. This bond may also have served as a source of social support, a factor that facilitates psychological well-being [3]. Following the advice of Colucci et al. [24] to consider patient-preferred interpreters in psychotherapy may prove beneficial. In contrast, non-completers' psychotherapists benefited less from the projects' resources, mentioning the manual and evidence-based treatment and willingness to treat UYRs as facilitators, possibly reflecting socially desirable behavior. In addition, it is possible that the psychotherapists felt less secure and thus preferred more guidance, which could be provided by the manual, even though five out of seven had worked with UYRs before.

*The challenges* reported by *completers’ psychotherapists and non-completers’ psychotherapists* showed that the challenges faced by completers’ psychotherapists do not necessarily lead to premature termination, but become more apparent with longer treatment duration, such as more contact with the Youth Welfare Office and more complex study documentation. In addition, the lack of educational background may become more pronounced during the cognitive processing module, which appears later in TF-CBT. Non-completers' psychotherapists also mentioned more structural challenges, such as the absence of a primary responsible caregiver and distant locations, as well as an increased effort for psychotherapists. This highlights the role of caregivers in successful trauma treatment and is consistent with research with primarily biological parents showing that their presence at the first session and adolescents' assessment of parents' treatment approval predicted treatment dropout [50]. An additional challenge for non-completers’ psychotherapists was the presence of an ongoing grief process. Given the prevalence of comorbid grief disorder and PTSS among refugees [51, 52], the integration of grief-specific treatment components is essential. The use of grief specific components during the TF-CBT treatment is also incorporated into the manual [7]. Research on the treatment of trauma and grief among UYRs is limited; however, a recent meta-analysis for children and adolescents suggests that grief-focused cognitive-behavioral psychotherapies are effective in reducing grief and related posttraumatic stress symptoms [53].

In summarizing key findings and offering practical implications, we have chosen to integrate our findings into Michaels et al.’s [54] “Socio-Ecological Model of Mental Health & Well-Being” which allows us to identify individual, organizational, and policy dimensions essential for successful trauma treatment. At the core of this model is the *individual*, the UYR, where treatment readiness and language proficiency serve as key facilitators. Subsequently, *interpersonal relationships* are a relevant factor, including the social support network, which comprises, for example, the CYWS facility and its caregivers. In this context, the support and compliance of the aforementioned individuals were of particular significance, as was their collaboration with the psychotherapist and their knowledge. For practical implications both the individual and interpersonal levels should be considered. It becomes evident that trauma-informed care is a recommended approach, which should include education and training for caregivers about symptomatology and trauma treatment. This will increase mental health literacy among caregivers and UYRs, and may result in greater mental health service use, treatment readiness, and higher treatment compliance among caregivers. These topics were all mentioned as facilitators within this study. At the *organizational* level, it is recommended that local partnerships are established between psychotherapists and CYWS facilities to decrease some of the structural challenges such as logistical, distance and coordination issues, which were also suggested by Borbon et al. [55] as part of their lessons learned for trauma-informed care with UYRs. At the *community* level, the use of skilled interpreters was seen as a facilitator and is recommended. Ideally, they are already known to the patient and can facilitate understanding of cultural differences, while building a trusting relationship with the patient and providing social support. Lastly, the *policy* makers have a huge responsibility, and our recommendation is the provision of resources, training and standards for CYWS facilities, psychotherapists and interpreters. CYWS need to be provided with the personal resources and above-mentioned training in order to be able to accompany treatment sessions and facilitate transportation, this is of particular importance, as the German federal states have lowered the minimum standards within UYR facilities [56]. Furthermore, we propose that psychotherapists get the ability to implement an outreach approach in collaboration with local partners. Finally, our recommendation is to implement country-wide requirements for interpreters in psychotherapy, ensuring adherence to data protection and confidentiality standards.

This study has several strengths, such as conducting interviews with twenty psychotherapists representing a range of backgrounds, experiences with UYRs, and regions across Germany. However, several limitations warrant further attention. First, it is possible that the experiences of the thirteen psychotherapists who had previously treated traumatized UYRs may have influenced the responses given in the present interviews. Second, although all invited psychotherapists participated, the sample is not representative of all psychotherapists because it only included those interested in treating traumatized UYRs. Third, the project funded treatment provided by two psychotherapists, as the UYRs did not yet have health insurance to cover the costs due to the ongoing asylum process. All psychotherapists had free access to German TF-CBT web training and were paid for documentation and case consultations. This arrangement may have led to socially desirable responses in the interviews. Fourth, the mode of interpreting (face-to-face, video, or telephone) may have influenced the triangular relationship and the development of a trusting relationship and thus the responses regarding interpreter-related facilitators and challenges need to be interpreted with caution. Fifth, the analysis of whether the reported facilitators and challenges differed between completers' psychotherapists and non-completers' psychotherapists should be interpreted with caution, as we considered only the completion status of patients and not the extent of symptom reduction or adherence to the manual.

It is recommended that future research include the perspectives not only of patients, but also of their caregivers and interpreters. It would be beneficial to gain further insight into how they perceive the patient-interpreter relationship. This could include whether they perceive challenges in an overly close relationship, or whether such perceptions differ between collectivist and individualist cultures. The possibility of further integrating interpreters into the therapeutic process may also be explored. In addition, the role of comorbid prolonged grief disorder symptoms in trauma-focused treatment requires further attention, with a recommendation to explore how they affect the efficacy of trauma treatment. In this regard, we also recommend an analysis of the adaptations that psychotherapists have made to the TF-CBT manual when working with UYRs in CYWS settings.

In conclusion, there are numerous facilitating factors for the psychotherapy of UYRs that should be addressed at the individual, interpersonal, organizational, community, and especially at the policy level. In particular, specialized training should be provided to psychotherapists on evidence-based treatment, to caregivers on the process and content of trauma-focused therapy, and to interpreters on effective collaboration and translation within treatment. Additionally, the supervision of psychotherapists and the compliance of caregivers are of immense importance.

## Supplementary Information

Additional file 1. Interview guide. The file comprises the original interview guide used in this study.

Additional file 2. Qualitative Results on the Worries before Participating in the Project. The file contains tables of codes, frequencies, and examples of worries that psychotherapists had before participating in the project.

Additional file 3. Qualitative Results of Project-Related, Structural, Personal, Patient-, and Interpreter-Related Facilitators and Challenges Treating Unaccompanied Young Refugees. The file includes tables with codes, frequencies, and examples of project-related, structural, personal, patient-related, and interpreter-related facilitators or challenges.

## Data Availability

The datasets used and analyzed during the current study are available from the corresponding author on reasonable request.

## Abbreviations

UYRs: Unaccompanied young refugees
CYWS: Children and Youth Welfare System
PTSS: Posttraumatic Stress Symptoms
TF-CBT: Trauma-Focused Cognitive Behavioral Therapy
