# Supplementary material for: Challenges and facilitators in treating unaccompanied young refugees with posttraumatic stress disorder in a dissemination trial: a qualitative study with psychotherapists
Source: Child Adolesc Psychiatry Ment Health. 2025 Mar 20;19:25. doi: 10.1186/s13034-025-00873-w (PMC11927342; doi:10.1186/s13034-025-00873-w)
Supplement: Supplementary file 1 — Additional file 1. Interview guide. The file comprises the original interview guide used in this study. [file 13034_2025_873_MOESM1_ESM.docx]

**ADDITIONAL FILE 1** **Interview guide**
