# Supplementary material for: Challenges and facilitators in treating unaccompanied young refugees with posttraumatic stress disorder in a dissemination trial: a qualitative study with psychotherapists
Source: Child Adolesc Psychiatry Ment Health. 2025 Mar 20;19:25. doi: 10.1186/s13034-025-00873-w (PMC11927342; doi:10.1186/s13034-025-00873-w)
Supplement: Supplementary file 2 — Additional file 2. Qualitative Results on the Worries before Participating in the Project. The file contains tables of codes, frequencies, and examples of worries that psychotherapists had before participating in the project. [file 13034_2025_873_MOESM2_ESM.docx]

**ADDITIONAL FILE 2: Qualitative Results on the Worries before Participating in the Project**

The categorization of structural, personal, and patient-related barriers, evident from the theoretical background, can also be applied to the worries previously reported by our sample before participating in our project as shown in Add2-Table 1. Five psychotherapists reported that they did not have any worries.

At the *structural level*, psychotherapists reported worries regarding organizational difficulties because UYRs live in child and youth welfare facilities and an uncertain residency status, as "[…] you never know how long they will stay in Germany. Is it worth it to actually start treatment?" (T14).

*Personal worries* were a high emotional strain due to the severe traumatization of UYRs, lacking cultural competencies and uncertainty in working with interpreters, which included questions such as "And would it be possible to get an interpreter […]" (T9).

*Patient-related worries* were lacking language proficiency, unreliable attendance and psychopathology more severe than typically seen in non-refugee youth, as "[…] there could be traumas that I am not used to from the normal everyday life here, more like things that might have existed here in the past and are [unintelligible] or more severe than what one normally encounters in practice at the moment." (T11).

Other worries mentioned were for example how to explain psychotherapeutic concepts in a child-friendly way and that “[…] this clientele is not exactly easy to integrate into care […]” (T17).

**Add2-Table 1**

*Former Worries in Treating Traumatized UYRs*

|  | Code | Frequency^a)^ | Example |
| --- | --- | --- | --- |
| No worries | | 5 (31.6%) | "No, not at all." (T2) |
| Structural worries | | | |
|  | Organizational difficulties | 4 (21.1%) | "[…] and then also regarding their insurance status. So, how do you do that if they don't have a card? How does it actually work?" (T9) |
|  | Uncertainty of the residence status | 3 (15.8%) | "One never knows how long they will stay in Germany. Is it worth it to actually start the treatment?" (T14) |
| Personal worries | | | |
|  | Emotional strain for psychotherapists | 4 (21.1%) | "Yes, I have already said, these fears that I can't bear the traumas they have experienced. That was another concern... especially with war experiences or so, I had not yet treated children and adolescents." (T4) |
|  | Uncertainty in working with interpreters | 4 (21.1%) | "And would it be possible to get an interpreter? So those were all kinds of questions that probably held me back beforehand." (T9) |
|  | Lacking cultural competences | 3 (15.8%) | "Then also the question, how well can I be culturally sensitive?" (T13) |
| Patient-related worries | | | |
|  | Language proficiency | 3 (15.8%) | "Of course, in terms of language, naturally. How good will the exchange be?" (T13) |
|  | Unreliable attendance | 2 (10.5%) | "[…]then there's always the question of reliability, if it were a concern, if one would want to name it as a concern. Or as a potential possibility, that it is increased." (T20) |
|  | Severe psychopathology | 2 (10.5%) | "[…]that there could be traumas that I am not used to from the normal everyday life here, more like things that might have existed here in the past and are [unintelligible] or more severe than what one normally encounters in practice at the moment." (T11) |
| Other worries | | 6 (31.6%) | “That was probably then that I would have said, yes, okay, when I think about it, this clientele is not exactly easy to integrate into care.” (T17) |

*Note.* ^a)^ Interviews with code *n* = 19
