# Supplementary material for: Challenges and facilitators in treating unaccompanied young refugees with posttraumatic stress disorder in a dissemination trial: a qualitative study with psychotherapists
Source: Child Adolesc Psychiatry Ment Health. 2025 Mar 20;19:25. doi: 10.1186/s13034-025-00873-w (PMC11927342; doi:10.1186/s13034-025-00873-w)
Supplement: Supplementary file 3 — Additional file 3. Qualitative Results of Project-Related, Structural, Personal, Patient-, and Interpreter-Related Facilitators and Challenges Treating Unaccompanied Young Refugees. The file includes tables with codes, frequencies, and examples of project-related, structural, personal, patient-related, and interpreter-related facilitators or challenges. [file 13034_2025_873_MOESM3_ESM.docx]

**ADDITIONAL FILE 3:** **Qualitative Results of Project-Related, Structural, Personal, Patient-, and Interpreter-Related Facilitators and Challenges Treating Unaccompanied Young Refugees**

**Add3-Table 1**

*Project-related Facilitators and Challenges*

| Project-related facilitators | | | Project-related challenges | | |
| --- | --- | --- | --- | --- | --- |
| Code | Frequency ^a)^ | Example | Code | Frequency ^b)^ | Example |
|  |  |  | No challenges | 8 (44.4%) | “No. I didn't really miss anything. It was good all around!” (T9) |
| Case Consultations |  |  | Case Consulations |  |  |
| General effectiveness | 12 (60.0%) | "So I found the case consultations very helpful." (T7) |  |  |  |
| Group-based learning | 8 (40.0%) | "Yes, so, as I said, exchange with colleagues." (T19) |  |  |  |
| Increases adherence | 7 (35.0%) | "Then she was always able to give good tips on what one can do to perhaps find one’s way back a little bit." (T4) |  |  |  |
| Enables flexibility within fidelity | 5 (25.0%) | "So, because I had a certain openness, I was actually able to do quite well. So it didn't bother me, because normally I would go into it much more. But that's why I had the case consultation. So, in a way, it always kept me somewhat within bounds. And because of that, I was able to cope with it well." (T2) |  |  |  |
| Reflecting about patient | 5 (25.0%) | "I think case consultation is where you can reflect on patients again. I find that helpful." (T18) |  |  |  |
| Supervising person as competent contact person | 4 (20.0%) | "[…] that I can also contact [the supervisor] […]" (T6) |  |  |  |
|  |  |  |  |  |  |
| Other case consultation-related facilitators | 6 (30.0%) | "Exactly! Easily accessible and on a very personal level, despite Corona and video." (T5) | Other case consultation-related challenges | 1 (5.0%) | "One thing comes to mind that I would find quite nice. But I believe this is again due to the time frame that it is not possible, if the case consultation could be made a bit more open, I would find that quite nice. That is, not only the individual communicates with the [supervisor], but that the others can also participate a bit... if they have questions or if they have suggestions or a feeling about it. That it's more of a group setting, instead of just being spectators while two talk to each other. I would wish for that a bit more, or I could express it as a point of criticism. I had also imagined it a bit differently, that it is more of a group supervision." (T4) |
| TF-CBT Workshop |  |  |  |  |  |
| General effectiveness | 4 (20.0%) | "The training itself was really cool, very. Also very memorable." (T8) |  |  |  |
| Conveys important knowledge | 4 (20.0%) | "Okay, I have now experienced in the workshop what it can look like." (T4) |  |  |  |
| Structure of the workshop | 2 (10.0%) | "The workshop, because it is summarized." (T2) |  |  |  |
| Other TF-CBT Workshop facilitators | 4 (20.0%) | "And then the workshop. It was fantastic. First of all, online, and then also just the two of us. I mean, it doesn't get much better than that." (T9) |  |  |  |
| Both case consultations and workshop | 5 (25.0%) | "So both completely. I found both totally helpful." (T3) |  |  |  |
| Material |  |  | Material |  |  |
| General availability of material | 12 (60.0%) | "Uh, so it was definitely one of the reasons to participate at all? For the, uh, for the materials? Because it's somehow difficult to get it standardized in any way." (T20) | Material not adequate | 3 (15.0%) | "To be honest, the therapy material, now in the form of worksheets, was less important for me and it's always very finely printed in a folder on the shelf, but it's not really helpful for my other patient group in principle. Yes, as a mnemonic, but not to pull out and use. And also for my patient. Since he was already 20 years old and knew a lot, I rather worked with materials from the adult sector or with materials I produced myself." (T5) |
| Availability of worksheets | 5 (25.0%) | "So whenever I thought I was floundering, I thought, okay, then I'll cling to what the worksheets provide." (T104) |  |  |  |
| Availability of translated materials | 5 (25.0%) | "I believe, of course, it was also fundamentally helpful that there was a French translation […]" (T14) |  |  |  |
| German TF-CBT Web |  |  | German TF-CBT Web |  |  |
| General effectiveness | 4 (20.0%) | "The online course was also certainly helpful. I think if you have never dealt with that before, then it would certainly be very helpful for many people." (T19) | Too extensive | 2 (10.0%) | "So the online training was more like so-so. Um... That's also the feedback, by the way, from other colleagues, that they just stopped after three percent, um..., because they thought, now I've been sitting here for two hours and it's three percent... that's more of a hurdle. I perceived it as a hurdle and I believe the others did too." (T8) |
| Video examples | 3 (15.0%) | "I also found these examples that you can look at quite good." (T9) |  |  |  |
| Repeated use possible | 2 (10.0%) | "What was helpful about the training itself was that you could always look into it again. So, for almost every session, before every module, I reviewed and read through the module again. Also, I watched one or the other video again. That was very helpful." (T4) |  |  |  |
| Flexibility | 2 (10.0%) | "Um, the structure, um this, yes, I can do it from home. I have my own pace and yes, can integrate it well into my daily routine." (T15) |  |  |  |
| Other German TF-CBT-Web-related facilitator | 1 (5.0%) | "The online learning then gave a first insight. Then you knew what it was about and also realized relatively quickly, okay, this is CBT, I recognize that somehow." (T121) | Other German TF-CBT-Web-related challenges | 2 (10.0%) | "I'm not much of an online learner. It annoys me." (T18) |
| Manualized and evidence-based treatment | 9 (45.0%) | "Okay, so this is, of course, a manual, so to speak. There are aspects, so to speak. It's about doing it just like that. And that's exactly it, no more and no less. So that is, that is, I find it quite okay again, because then, both I and the therapist, uh the patient knows what he is getting, so to say." (T1) | Documentation | 7 (35.0%) | "The only thing that was sometimes a bit difficult for me was this questionnaire that you were supposed to fill out after the sessions because with the options that were there, I sometimes didn't know: What do I click now?" (T12) |
| Provision / Funding of interpreters | 8 (40.0%) | "[…] but also about interpreters, so that I don't have to worry about it, because it's just more effort that I can't even manage in everyday life." (T14) | Lacking information on organizational aspects | 4 (20.0%) | "So personally, I occasionally got mixed up with the study design. So, who does the screening? Where do you get the questionnaire? Who evaluates it? It was a bit... opaque for me." (T7) |
| Financial compensation for therapists | 8 (40.0%) | "Yes, it was definitely an additional incentive to do it. That this extra effort is somehow also paid for. Because I believe that this is something I've heard in discussions with colleagues, that many are discouraged from working with refugees because this networking is just terribly annoying. And that no one pays you for it. I mean, during Corona, it wasn't so bad because you could make phone appointments, but once that was abolished, no one really felt like it anymore." (T7) | Knowledge decacy | 2 (10.0%) | "I just found it a bit difficult until the first patients arrived. Naturally, you then slip back into your daily routine, because, yes, I also treated many patients and then that part slips away again. I found that a shame, because it then required more effort from me, so to say." (T2) |
| Preparation and Initiation of treatment | 7 (35.0%) | "[…] through the making of contact and assignment of patients, this greatly eases the situation." (T5) |  |  |  |
| Availability of contact persons | 5 (25.0%) | "[…] and as I said, you could always ask [via email] if anything was missing and certainly the contact with the study center as well." (T19) |  |  |  |
| Digital implementation | 3 (15.0%) | "I found it perfect that everything was online. So for me, it was something special." (T9) |  |  |  |
| Other project-related facilitators | 5 (25.0%) | "Of course, without BetterCare it wouldn't have happened, and it's somehow, I would say nice, to be part of such a network. So, I benefit from it too and everything that is done about it." (T15) | Other project-related challenges | 4 (20.0%) | "It made things a bit more confusing. Hm, otherwise, yes, also, I mean, it's good that when it was said in supervision 'Ah, I have another article,' that it was forwarded, but I believe it would have been clearer, I think, to place it as supplementary material on the central server somehow. And then, for example, that it has a uniform labeling. What it exactly is, because I then somehow had 15 emails, and materials as discussed and then you clicked through and then it was labeled AB 57 11 and you were like 'hmm?'." (T7) |

*Note.* ^a)^ Interviews with code *n* = 20; ^b)^ Interviews with code *n* = 18

**Add3-Table 2**

*Structural Facilitators and Challenges*

| Structural facilitators | | | Structural challenges | | |
| --- | --- | --- | --- | --- | --- |
| Code | Frequency ^a)^ | Example | Code | Frequency ^b)^ | Example |
|  |  |  | No challenges | 6 (30.0%) | "There was nothing." (T10) |
| CYWS facility aspects |  |  | CYWS facility aspects |  |  |
| Supporting/accompanying the treatment sessions* | 7 (38.9%) | "Yes, it was always the same primary caregiver involved, except when she was sick or had something else." (T11) | Lacking clear primary clearly responsible caregiver* | 7 (35.0%) | "I found this collaboration with the home incredibly exhausting and also frustrating. So again, a reliability issue with the contact person." (T13) |
| High treatment compliance from caregivers/facility* | 8 (44.4%) | "[…] definitely the willingness of the institution to participate, I believe" (T20) | Lacking therapy compliance from caregivers/facility* | 3 (15.0%) | "Yes, they were not thrilled and are not thrilled about the treatment and they were always very uncooperative." (T9) |
| Supportive caregivers in everyday life | 5 (27.8%) | "Yes, but. And therefore, because they had a good caregiver network, I didn't have to worry about things like school, residence permits, or anything else, but I could focus on the therapy." (T104) | Caregiver-imposed outcome pressure | 2 (10.0%) | "Youth welfare facility, okay, so that sometimes educators are in a total crisis and then hope for quick help from the therapist, kind of like this 'fix it quickly so things run smoothly'." (T18) |
| Collaboration and exchange between caregivers and psychotherapist* | 3 (16.7%) | "That the exchange went so smoothly and always timely, both with the facility where he was accommodated." (T15) | Lacking caregiver/conjoint sessions* | 4 (20.0%) | "Of course, it was still complicated because the service and shift schedule often meant that we couldn't have a joint session, because he was not on duty, didn't bring him, um yes exactly." (T121) |
| Knowledge about PTSD/psychotherapy* | 2 (11.1%) | "That they recognize it themselves, so the caregivers, that they recognize, that they understood well, what traumatization was." (T2) | Lacking knowledge about PTSD/psychotherapy* | 3 (15.0%) | "[…] and I actually didn't really get the impression, that's my impression, understood anything. Nonsense. The impression that she understood what traumatization is like in everyday life and also when I did even more translation work. I didn't get the impression that it was received that way." (T2) |
| Facilitating transportation* | 2 (11.1%) | "So, I found that in the facility... it often didn't work out well for them to participate in person, but they really made an effort to ensure that he... that he was taken to his appointments, so that he was picked up. They always registered in advance. So, the facility was very committed and I believe it also made things easier." (T3) | Logistical access issues* | 4 (20.0%) | "With the facility, there were isolated, so minor difficulties, isolated cases where they couldn't drive him, then somehow to an appointment or something, and that's why it was canceled" (T121) |
| Other facility related facilitators | 2 (11.1%) | "I liked about the caregivers that the facilities had a very clear information system in the house, that they did it like hospitals and kept the files online, and no matter who you talked to, they knew exactly what had been discussed with the previous person. Which is not a matter of course with the facilities." (T7) | Other facility related challenges | 6 (30.0%) | "Hm, so, youth welfare, just like, these youth welfare structures, so from shift work, absences, difficulties in reaching, that's what I found" (T13) |
| Availability of interpreters | 9 (50%) | "Well, it would have been impossible without an interpreter for the one; he spoke such poor German that no therapy could have been conducted by me. He couldn’t speak English either. That's another thing with language, and there a treatment would have been impossible." (T10) | Long distance between facility & psychotherapy | 7 (35.0%) | "The second difficulty was the long drive time." (T1) |
| Use of supplemental materials | 4 (22.2%) | "Occasionally perhaps one other booklet, to address the topic of feelings in more detail, especially at the beginning. I would say I brought that in, content-wise. Otherwise, I used almost all the materials from the project." (T14) | Difficult time coordination | 8 (40.0%) | "So, organizationally, it was difficult to synchronize the interpreters' schedule and mine, and if it didn't work out on a certain day, we usually didn't find any alternative dates. Because we were both fully booked and it just didn't work out." (T7) |
| Location of the practice | 2 (11.1%) | "Or that the practice is perhaps relatively central. The thing with the train station, I believe, is also quite good. So, those are indeed factors. Okay, and I have here also a whole, whole lot of stuff." (T1) | Increased effort | 6 (30.0%) | "Yes, and that actually took up a lot of time beforehand, constantly calling to explain the situation. And in the end, I also worked an hour longer than I actually wanted to work that day. So I'm not used to having to promote patients in advance or to... yeah, to persuade patients like that […]" (T9) |
|  |  |  | Technical issues | 4 (20.0%) | "So if anything, it was more of a technical nature, like when the internet would go on strike, the program?" (T14) |
|  |  |  | Lacking clear responsibility from Youth Welfare Office | 4 (20.0%) | "And I notice it now too... I have to clarify it in advance. But nothing comes back from there. Like, how often I have called and sent emails. In every phone call, I'm put off, referred to someone else. It's really, really difficult." (T4) |
|  |  |  | Unreliability of public transportation | 2 (10.0%) | "It's like this, the train arrives later now. Yes, then we only have the 30 minutes or I do. Or if I have time afterwards, then we'll just go a bit longer." (T1) |
| Other structural facilitators | 5 (27.8%) | "The approval was really quick and completely hassle-free, and for the first one, he then started training, and so the youth welfare office practically handed it over to the health insurance. And then I just got the remaining hours approved by the health insurance. That was totally easy, totally relaxed. It went really well." (T3) | Other structural challenges | 6 (30.0%) | "So what made it difficult with this one patient was that his asylum application was rejected, even though it really looked promising. That made a huge difference. And what the subsequent issues are, how he will be treated in the future, which is, oh, always bogged down by bureaucracy, what he is allowed to do at all, nonsensically, yes, which school he can attend, which not, and so on, even though he is very committed. And then at some point, they are in such a phase. There was nothing you could do." (T2) |

*Note.* ^a)^ Interviews with code *n* = 19; ^b)^ Interviews with code *n* = 20; * Alignment of the Codes Facilitators - Challenges

**Add3-Table 3**

*Personal Facilitators and Challenges*

| Personal facilitators | | | Personal challenges | | |
| --- | --- | --- | --- | --- | --- |
| Code | Frequency ^a)^ | Example | Code | Frequency ^b)^ | Example |
| Good therapeutic alliance | 6 (46.2%) | "And that... that a relationship, a therapeutic relationship, could develop there, in which I also felt comfortable." (T4) |  |  |  |
| Openness to treat UYRs | 3 (23.1%) | "But on the other hand, I also like treating refugees! But it might make things a bit easier, indeed." (T1) |  |  |  |
| Other personal facilitators | 6 (46.2%) | "Well, I already knew the caregiver before BetterCare and the interpreter as well, and yes, it was just a good foundation that we had already established beforehand." (T12) | Other personal challenges | 5 (100.0%) | "So, I can be a bit impatient sometimes, I then have to hold myself back and say, maybe this isn't the topic for today after all. We need to take a step back again." (T15) |

*Note.* ^a)^ Interviews with code *n* = 13; ^b)^ Interviews with code *n* = 5

**Add3-Table 4**

*Patient-related Facilitators and Challenges*

| Patient-related facilitators | | | Patient-related challenges | | |
| --- | --- | --- | --- | --- | --- |
| Code | Frequency ^a)^ | Example | Code | Frequency ^b)^ | Example |
| Treatment readiness* | 6 (54.5%) | "Yes, and I would say, the patient's own will as well." (T15) | Lacking treatment readiness* | 5 (33.3%) | "We then revisited the symptoms, yes, I believe, this concept of allowing oneself to be helped was not yet acceptable to him. He was not at that point yet." (T18) |
| Language proficiency* | 2 (18.2%) | "And of course, it was good that she spoke German well. I don't know if it would have worked the same with a significant language barrier." (T9) | Lacking language proficiency* | 9 (60.0%) | "I actually found the language barrier difficult, even though, of course, an interpreter could have been used, but there was no acceptance on the part of the patient, at least at that time." (T13) |
|  |  |  | Concerns regarding family | 6 (40.0%) | "I believe that he somehow needs certainty over this 'my family is coming or not coming' in order to either deal with the fact that they are not coming, or if they do come, then the traumatic issues will resurface for him. At the moment, all of this doesn't come up at all, it has no meaning for him." (T3) |
|  |  |  | Complex daily challenges | 4 (26.7%) | "Uh, yes. Um, I would say, uh the, the turbulence of their everyday lives. Yes. Youth welfare and trauma have the potential to make everyday life unstable." (T20) |
|  |  |  | Grief | 2 (13.3%) | "I believe my patient was still in mourning, thus it was less about the traumatization, which he definitely had, but also this grief and being torn away from a somewhat good life." (T18) |
|  |  |  | Lacking educational background | 2 (13.3%) | "So I couldn't always use all of them [worksheets]. It becomes very difficult with this case because he is illiterate. We'll have to come up with something." (T4) |
| Other patient-related facilitators | 9 (81.8%) | "So the first therapy is... it's still ongoing, and it really went by the book, I would say. Also because there were and are really great conditions with that boy. And I thoroughly enjoyed it too." (T3) | Other patient-related challenges | 9 (60.0%) | "Mhm, yes, with one of them it was really in that trauma, this jumping around, where he had to be very narrowly confined, when you say 'No, no, no, no, we are only in Iran, we are now only dealing with this story on this mountain,' and that otherwise the stories would become endless. That made it difficult. But otherwise, it actually went quite well." (T7) |

*Note.* ^a)^ Interviews with code *n* = 11; ^b)^ Interviews with code *n* = 15; * Alignment of the Codes Facilitators - Challenges

**Add3-Table 5**

*Interpreter-related Facilitators and Challenges*

| Interpreter-related facilitators | | | Interpreter-related challenges– | | |
| --- | --- | --- | --- | --- | --- |
| Code | Frequency^a)^ | Example | Code | Frequency^b)^ | Example |
| Precise word-for-word translation* | 6 (40.0%) | "Overall very positive, because the interpreter I had, Mr. J., really translated well and, uh, I always had the impression that he was translating one-to-one, […]" (T12) | Lacking word-for-word translation* | 6 (60.0%) | "And also, that she manages to translate word for word. I really struggled with this, and at some point, it severely disrupted the flow of therapy... disturbed, when I again said, 'Please, Mrs. G., remember to do it like this,' and the next time she does it again anyway […]" (T104) |
| Transparency | 6 (40.0%) | "He did that very well, I had the impression, he also always asked again and said, when he mentioned: well, you know, this and that in the language is difficult to explain. May I, so, he also asked, even if it went beyond the translation, […]" (T18) | Own therapeutic needs | 4 (40.0%) | "I've had negative experiences with interpreters who, let's say, due to cultural reasons, had their own issues. Now, also in the context of political, cultural topics, [inaudible: now let's say with the development of the Taliban locally, it happened that for some interpreters, there was a bit of a change, it's quite difficult with these interpreters, the situation, the traumatic situation]." (T6) |
| Trusting bond with patient | 6 (40.0%) | "And also here, so to speak, the human aspect, meaning that one could tell that the young people also liked talking with this interpreter. That was also very important. And they actually treated him like an uncle." (T7) | Relationship with patient too close | 3 (15.0%) | "Another difficulty is that I get the impression that some young people tend to bond more with the interpreter than with me." (T104) |
| Caring/likeable interpreter | 6 (40.0%) | "[…] it was important to her as well. So that she was like... So she wasn’t a businesswoman in that sense, but she participated, truly participated with her heart. And that... She then took her time, whenever it was necessary. That was certainly another factor." (T4) |  |  |  |
| Experienced/Trained interpreter | 5 (33.3%) | "[…] where it then got better with the new interpreter, who was simply better trained in the program, […]" (T11) |  |  |  |
| Cultural mediator | 4 (26.7%) | "[…] indeed, a cultural bridge was built, was built. Yes, then, for instance, somehow the young person mentioned an idiom in the narrative, something with the Angel of Death Isarel or something, and then she somehow explained to me, yes, in Afghanistan it's like this and that, and it means this and that, so to speak, somehow, you know. That was positive, clearly, that was also a form of cultural mediation alongside the linguistic, I would say. Uh, exactly." (T121) |  |  |  |
| Language mediator | 3 (20.0%) | "I would like to say that again, I believe he really translated well. That's not the issue here.". (T10) |  |  |  |
| Remote interpreter | 3 (20.0%) | "And, uh, and then the...the...the switch to the online interpreter was better because, due to, because the computer was there, it was a bit more in the background." (T11) | Remote interpreters | 3 (15.0%) | "The difficult part was that he was only available by phone, so to speak. I didn't find that very helpful. I do think it's good to be able to see the person. So, it wasn't good that he didn't manage to do it with a phone [means: video]." (T1) |
| Long-term continuity | 2 (13.3%) | "But that's probably also because he conducted the group conversations and everything. So, basically, he did everything for this institution except for the BAMF [BAMF stands for "Bundesamt für Migration und Flüchtlinge," which translates to the Federal Office for Migration and Refugees]." (T7) | Interpreters wish to act as co-therapist | 2 (10.0%) | "Or when they did not just perform their task but tried to act therapeutically themselves or became too emotional" (T6) |
| Other interpreter-related facilitators | 7 (46.7%) |  | Other interpreter-related challenges | 7 (70.0%) | "Uh, as I said, it was difficult at times because he would cancel appointments at short notice, and that's just inconvenient in private practice when someone cancels ten minutes before. Then I have a loss that I can't charge the young people for, even if he... I wouldn't have done that. But I did consider charging him for it at times. And then there were two clarifying conversations. Once I spoke with him alone and once with the caretaker from, uh, from the institution, and after that, things went smoothly." (T12) |

*Note.* ^a)^ Interviews with code *n* = 15; ^b)^ Interviews with code *n* = 10; * Alignment of the Codes Facilitators - Challenges
